# Clinical significance of KAI1/CD82 protein expression in nasopharyngeal carcinoma

**DOI:** 10.3892/ol.2015.2891

**Published:** 2015-01-23

**Authors:** GENGMING WANG, HAO JIANG, HONGBO XU, QIAN SUN, YAN ZHOU, PING XIANG, ZENONG CHENG, YAJUN ZHANG, YUFU ZHOU, QING GUO, XINGLONG DU, SHUXIU XU, SHIYIN MA, ZHENDONG CHEN

**Affiliations:** 1 Department of Oncology, The Second Affiliated Hospital of Anhui Medical University, Hefei, Anhui 230601, P.R. China; 2 Department of Radiotherapy, The First Affiliated Hospital of Bengbu Medical College, Bengbu, Anhui 233004, P.R. China; 3 Central Laboratory, The First Affiliated Hospital of Bengbu Medical College, Bengbu, Anhui 233004, P.R. China; 4 Department of Pathology, The First Affiliated Hospital of Bengbu Medical College, Bengbu, Anhui 233004, P.R. China; 5 Department of Radiotherapy, Taizhou People’s Hospital, Taizhou, Zhejiang 225300, P.R. China; 6 Department of Radiotherapy, The Second People’s Hospital of Chuzhou, Chuzhou, Anhui 239000, P.R. China; 7 Department of Otolaryngology, The First Affiliated Hospital of Bengbu Medical College, Bengbu, Anhui 233004, P.R. China

**Keywords:** expression, nasopharyngeal carcinoma, KAI1/CD82, protein, metastasis

## Abstract

The aim of this study was to investigate KAI1/CD82 protein expression in human nasopharyngeal carcinoma (NPC) cell lines and human NPC tissues. Immunohistochemistry and western blot analysis were used to detect the localization and expression levels of the KAI1/CD82 protein in five human NPC cell lines. Immunohistochemistry was also conducted to detect the expression of the KAI1/CD82 protein in 70 NPC tissues and 30 non-neoplastic nasopharyngeal tissues. The levels of KAI1/CD82 protein expression were found to decrease as the metastatic potential of cells increased. The expression rate of KAI1/CD82 protein in the NPC tissues (44.3%) was significantly lower than that in the non-neoplastic nasopharyngeal tissues (70.0%) (P<0.05). KAI1/CD82 protein expression in the NPC tissues was not associated with clinical parameters, including gender, age, histological type and T stage, and the positive expression of KAI1/CD82 decreased with increased N staging. The level of KAI1/CD82 protein expression was increased in different human NPC cell lines. The KAI1/CD82 gene was highly expressed in cells with low metastatic potential, while low expression was observed in cells with a high metastatic potential. In addition, the KAI1/CD82 gene was expressed at low levels in nasopharyngeal carcinoma tissues, while high expression was identified in non-neoplastic nasopharyngeal tissues, and was associated with lymph node metastasis. These results indicated that the KAI1/CD82 gene may be involved in the occurrence, development and metastasis of nasopharyngeal carcinoma.

## Introduction

Nasopharyngeal carcinoma (NPC) is a highly invasive malignant tumor, which occurs in the nasopharynx. Although it is rare in the majority of countries (1), the incidence and mortality rates of NPC are markedly high among Southern Chinese populations. The five-year survival rate following combined treatment with radiotherapy and adjuvant cisplatin chemotherapy is 50–60% and the rates of five-year cumulative local relapse and distant metastasis are 20–30 and 20–25%, respectively (2). Therefore, investigating distant metastasis in NPC, and minimizing the occurrence of distant metastasis have become key to further improve the efficacy of NPC treatment and, thus, have important practical significance. The KAI1/CD82 gene, which belongs to the transmembrane 4 superfamily (TM4SF), was identified by Dong *et al* (3) in 1995. The inhibitory effects of the TM4SF protein on tumor metastasis have been demonstrated (4); its cell-membrane location and extensive glycosylation leads to cell-cell and cell-extracellular matrix interactions, which subsequently affect tumor metastasis. These interactions are extremely important with regard to the invasion and metastasis of tumors. In this study, immunohistochemistry and western blot analysis were used to detect the levels of KAI1/CD82 protein expression in five different human NPC cell lines, CNE-1, CNE-2Z, SUNE-1, SUNE-1-5-8F and SUNE -1-6-10B, and immunohistochemistry was also performed to detect the KAI1/CD82 protein expression in NPC and non-neoplastic nasopharyngeal tissues. The association between abnormal KAI1/CD82 gene expression in NPC and patient age, gender, histological type, T staging and lymph node staging were analyzed. The aim of this study was to investigate novel methods, which may improve the treatment efficacy and the prognosis, as well as reduce the occurrence of metastasis.

## Materials and methods

### Specimens

The human NPC cell lines, CNE-1, CNE-2Z, SUNE-1, SUNE-1-5-8F and SUNE -1-6-10B, with various metastatic characteristics were purchased from Hefei Shengmai Reagent Co., Ltd (Hefei, China). The detailed metastatic characteristics and levels of differentiation are shown in Table I.

A total of 70 archived paraffin-embedded NPC specimens were obtained from the Department of Pathology. The First Affiliated Hospital of Bengbu Medical College (Bengbu, China) between February 2007 and August 2010. The clinical data of all patients were complete and no patients had received radiotherapy or chemotherapy prior to biopsy. In addition, 30 archived paraffin-embedded non-neoplastic nasopharyngeal tissue specimens served as the control group, which were all samples of nasopharyngeal chronic mucosal inflammation, with or without the lymphoid hyperplasia. This study was conducted in accordance with the Declaration of Helsinki and with approval from the ethics committee of The First Affiliated Hospital of Bengbu Medical College. Written informed consent was obtained from all participants.

### Immunohistochemical detection of KAI1/CD82 expression in human NPC cell lines

After anabiosis, medium-changing and three passages, the five NPC cell lines were seeded in the pre-sterile-coverslip-paved six-well plates at a density of ~5×10^4^ cells/ml. Following incubation at 37°C in an atmosphere of 5% CO_2_ for 48 h, the coverslips were removed and immunohistochemical staining with Histostain™-Plus kits (Beijing Zhongshan Biotechnology Co., Ltd., Beijing, China) was performed at room temperature. The appearance of brown granules on the cell surface and inside the cytoplasm was considered to indicate positive KAI1/CD82 expression. A total of four cell-attached coverslips of the KAI1/CD82 protein from each cell line were randomly selected, and 500 cells in each cell-attached coverslip were randomly selected under a microscope (BX50; Olympus, Tokyo, Japan; magnification, ×400); the total number of cells in the four cell-attached coverslips was 2,000. The number of positive cells among the 500 randomly selected cells in each of the above four cell-attached coverslips was counted to calculate the positive KAI1/CD82 expression rate, with the following formula: Positive rate of KAI1/CD82 expression (%) = number of positive cells / total number of cells counted. According to the number of cells and the percentage of positive cells, the results were classified into four grades: No expression (−), no positive cells in the cell-attached coverslip; low expression (+), >0% and <24% positive cells; moderate expression (++), 25–50% positive cells; and high expression (+++), >50% positive cells.

The χ^2^ test was used to compare the positive rates of KAI1/CD82 protein among the cell line with the lowest metastatic potential (SUNE-1-6-10B) and the other cell lines with higher metastatic potentials.

### Western blot analysis

A total of 1×10^7^ cells of each of the five NPC cell lines, which were wall-adherent, were collected. Next, 200 μl cell lysate was added for the 30 min lysis on ice, followed by centrifugation at 2,862 × g for 30 min at 4°C (5810R, Eppendorf, Hamburg, Germany). The supernatant was obtained, and the protein concentration was determined using the Coomassie Brilliant Blue assay (G-250, Beijing Zhongshan Biotechnology Co., Ltd.). The protein concentration was then adjusted to 50 μg/μl. Next, 10% SDS-PAGE electrophoresis was performed for 3 h to separate the proteins, which were then transferred to nitrocellulose membranes. After three washes with phosphate buffered-saline (PBS; Fuzhou Maixin Biotechnology Development Co., Ltd., Fuzhou, China) for 15 min each, l% bovine serum albumin (Fuzhou Maixin Biotechnology Development Co., Ltd.) was used to block non-specific antigen activity for 2 h. After blocking, the membranes were incubated with mouse anti-human KAI1/CD82 monoclonal primary antibody (l:250; sc-17752; Santa Cruz Biotechnology, Inc., CA, USA) overnight at 4°C, followed by washing with PBS. The alkaline goat anti-rat polyclonal phosphatase-labeled secondary antibody (l:200; PV6002, Beijing Zhongshan Biotechnology Co., Ltd.; general-type kit; Fuzhou Maixin Biotechnology Development Co., Ltd.) was added, followed by incubation at room temperature for 2 h. NBT/BCIP coloration was conducted for 15 min (kits were provided by Fuzhou Maixin Biotechnology Development Co., Ltd., Fuzhou, China). When clear brown bands, which indicate positive staining, were observed on the membranes, the coloration was terminated, followed by rinsing, drying and preservation of the membranes.

### Immunohistochemical detection of KAI1/CD82 protein expression

All NPC and non-neoplastic nasopharyngeal tissue specimens were fixed in 10% formalin, embedded in paraffin and cut into 4 μm-thick serial sections. After dewaxing and hydration, antigen retrieval in potassium citrate buffer was performed on the sections using a microwave. Horseradish peroxidase (Fuzhou Maxim Bioengineering Co. Ltd., Fuzhou, China) was then added to label the avidin, followed by staining with 3,3′-Dimethylbenzidine (Fuzhou Maixin Biotechnology Development Co., Ltd.). After rinsing with water, the specimens were re-stained with hematoxylin (Fuzhou Maixin Biotechnology Development Co., Ltd.), followed by an additional rinse with water. The specimens were then dehydrated in ethanol and cleared in xylene (Wuxi Jingke Chemical Co., Ltd., Wuxi, China), followed by mounting with neutral gum (Fuzhou Maixin Biotechnology Development Co., Ltd.). PBS was used to replace the primary antibody for the blank control group. The nasopharyngeal tissues were observed under a microscope (BX50; Olympus), and the appearance of brown particles in the cytoplasm was considered to indicate positive expression. A total of four different views at high magnification were performed to count 100 cells in each view, which were divided into (+) to (+++) according to the percentage of positive cells, which was scored as follows: no positive cells (−); 1–9% positive cells (+); 10–50% positive cells (++); >50% positive cells (+++). The (+) to (+++) scores were classified as positive expression, while (−) score was considered as negative expression.

### Statistical analysis

The western blotting data were analyzed using one-factor analysis of variance and the immunohistochemistry data were analyzed by the χ^2^ test. All data were analyzed using SPSS software, version 17.0 (SPSS, Inc., Chicago, IL, USA) and P<0.05 was considered to indicate a statistically significant difference.

## Results

### Immunohistochemical detection of KAI1/CD82 protein levels in the five NPC cell lines

**Figure 1:**
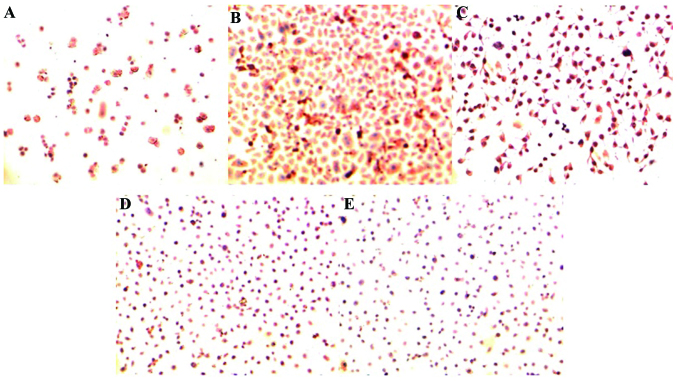
KAI1/CD82 protein expression in human nasopharyngeal carcinoma in (A) SUNE-1-6-10B, (B) CNE-1, (C) CNE-2Z, (D) SUNE-1 and (E) SUNE-1-5-8F cell lines with different metastatic potentials (magnification, ×100).

The immunohistochemical results showed that the KAI1/CD82 protein was significantly expressed in the membrane and/or cytoplasm of all five NPC cell lines, with the positive signal of brown particles. The results revealed that the KAI1/CD82 protein was located in the membrane and/or cytoplasm in all five cell lines. KAI1/CD82 was highly expressed in the cytoplasm and membrane of the SUNE-1-6-10B cell line (tumorigenesis and low metastatic potential), while low expression was exhibited in the cytoplasm and membrane of the SUNE-1-5-8F cell line (high tumorigenesis and metastatic potential). In the remaining cell lines, the expression varied (Fig. 1). The positive rate of KAI1/CD82 protein expression in each cell line was calculated and the χ^2^ test was performed, which revealed that the positive expression rate of KAI1/CD82 protein in the SUNE-1-6-10B cells (tumorigenesis and low metastatic potential) was significantly higher than that in the cell lines with a higher metastatic potential (P<0.01).

SPSS 17.0 software was used to perform the completely randomized-design χ^2^ test, which revealed that the positive expression rate in the SUNE-1-6-10B cells was significantly different when compared with the other groups (P<0.05) (Table II). Furthermore a significant difference in expression rate was identified between the CNE-1 and SUNE-1 cell lines, and between the CNE-2Z and SUNE-1-5-8F cell lines (P<0.05), while no significant difference was identified between the SUNE-1 and CNE-2Z cell lines (P>0.05).

### Western blot analysis of KAI1/CD82 protein levels in the five NPC cell lines

**Figure 2:**
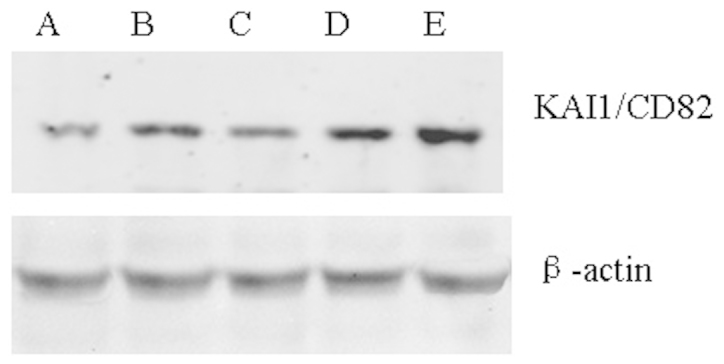
KAI1/CD82 gene expression in different human nasopharyngeal carcinoma cell lines. (A) SUNE-1-5-8F, (B) CNE-2Z, (C) SUNE-1, (D) CNE-1 and (E) SUNE-1-6-10B.

Western blot analysis revealed that the KAI1/CD82 protein expression levels differed in the five NPC cell lines (Fig. 2). β-actin was used as the internal reference. The results revealed that compared with the SUNE-1-5-8F cell line, the protein expression of KAI1/CD82 in the SUNE-1-6-10B and CNE-1 cell lines was significantly increased (P<0.05) (Table III).

SPSS 17.0 software was used to perform the LEVENE homogeneity of variance test, the results of which were F=0.485 and P=0.747 (P>0.05), indicating that the data of the five groups exhibited homogeneity of variance. Furthermore, the completely randomized-design analysis of variance results were P=0.000 (P<0.05) and F=315.775; therefore, it was considered in general that the data obtained from the five groups were different. The pairwise comparison with least significant difference and Student-Newman-Keuls methods identified no significant difference in the protein expression between the CNE-2Z and SUNE-1 cell lines (P=0.195 and P>0.05, respectively); however, the pairwise comparisons between the other groups identified a significant difference (P<0.05).

### Expression of the KAI1/CD82 protein in NPC and non-neoplastic nasopharyngeal tissues

**Figure 3:**
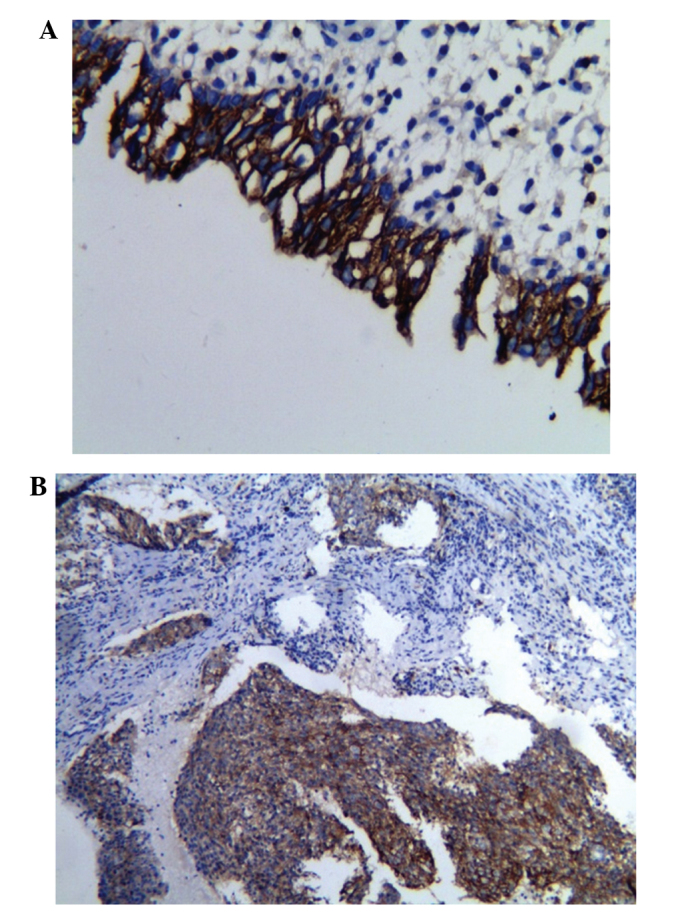
Positive KAI1/CD82 protein expression was identified in (A) non-neoplastic nasopharyngeal and (B) NPC tissues by immunohistochemistry. (magnification: A, ×40; B, ×10).

The immunohistochemical detection of the KAI1/CD82 protein expression in the two groups revealed that the positive expression rate of the KAI1/CD82 protein in the non-neoplastic nasopharyngeal tissues (21/30; 70.0%) was significantly higher than that of NPC group (31/70; 44.3%), and the χ^2^ test revealed a statistically significant difference in KAI1/CD82 protein expression between the two groups (Fig. 3) (P<0.05).

### Association between KAI1/CD82 protein expression and clinical factors

The expression of KAI1/CD82 protein in NPC was not found to correlate with the clinical characteristics of patients, including age, histological type and T staging, which was determined according to the International Union Against Cancer’s (UICC) TNM staging system (5). However, the expression of KAI1/CD82 protein expression was found to correlate with lymph node metastasis. The positive expression rate of KAI1/CD82 protein in patients without lymph node metastasis (N_0_), according to the UICC TNM staging system (5), was 68.4% (13/19), which was higher than that (35.3%, 18/51) in the patients with the cervical lymph node metastasis (N_1–3_), and this difference was statistically significant (P<0.05). With increased N staging, the KAI1/CD82 protein expression decreased (N_0,_ 68.4%; N_1_, 43.8%; N_2_, 33.3%; and N_3,_ 25.0%) (Table IV).

## Discussion

Metastasis refers to the process by which malignant cells detach from the primary tumor and migrate to a secondary tissue or organ via a variety of mechanisms. The cells continue to proliferate and grow, finally forming a secondary tumor that exhibits the same features as the primary tumor. Metastasis is one of the basic biological characteristics of malignant tumors, and the reason for the failure of treatment in the majority of cancer patients. Therefore, controlling metastasis is the key to improving the prognosis of cancer patients (6).

The KAI1/CD82 gene is located on the human chromosome 11p11.2 (3), which consists of 10 exons and nine introns (~80 kb) (3). The structure of the product of the KAI1 gene is the same as CD82 and, thus, KAI1 is a member of the TM4SF family. The KAI1/CD82 gene exhibits an inhibitory function with regard to tumor metastasis, and this inhibition has been confirmed in a number of studies investigating malignant cancer (7–10). However, this inhibitory mechanism remains unclear, and previous studies have shown that multiple factors are involved, which may exert effects on their own or in combination, including the integrin family (11), epidermal growth factor receptor (12), EW12/PGRL (13), KITENIN (14,15) and protein kinase C (16).

An initial study indicated that the KAI1/CD82 gene exhibited the inhibition specifically towards the metastasis of prostate cancer (17); however, later studies have revealed that the inactivation of this gene occurs in a number of other malignant tumors, including thyroid (14), breast (18–20), endometrial (16), laryngeal (21), colon (22), gastric (7,23), gallbladder (24), liver (25), kidney (26), bladder (27) and prostate (28) cancer. In addition, these studies also demonstrated that the inactivation of the KAI1/CD82 gene was associated with tumor metastasis.

Although the KAI1/CD82 gene has been reported to be involved in numerous other cancer types, its involvement in NPC is largely unclear. In the present study, immunohistochemistry and western blot analysis were performed to investigate the expression levels of the KAI1/CD82 gene in five different NPC cell lines, which exhibited different metastatic characteristics. The KAI1/CD82 protein levels were found to correlate with the metastatic characteristics of the NPC cell lines. The positive expression rate of KAI1/CD82 in NPC was lower than that in the normal nasopharyngeal tissues, indicating that the KAI1/CD82 gene may be involved in the occurrence and development of NPC. In NPC, the underexpression of the KAI1/CD82 protein was found to correlate with lymph node metastasis. Furthermore, with the progression of N staging, the expression rate of KAI1/CD82 protein was found to gradually decline, indicating that the underexpression of KAI1/CD82 protein was associated with NPC metastasis, and that KAI1/CD82 may be involved in the metastasis of NPC.

Controlling tumor metastasis is a major focus of cancer research; tumor metastasis is the main reason for treatment failure and patient mortality, and there are currently difficulties with regard to the treatment of malignant cancer. Therefore, future studies investigating the tumor metastasis-related genes are required to improve the efficacy of tumor treatment. Since the follow-up period in the present study was short, the association between KAI1/CD82 gene expression and the treatment and prognosis of NPC was not analyzed. Further study is required to elucidate the true association between KAI1/CD82 expression and the biological behaviors of NPC.

## Figures and Tables

**Table I:** Differentiation status and metastatic characteristics of the five cell lines used in this study.

Cell line	Level of differentiation	Metastatic characteristics
SUNE-1	Poorly differentiated	SqCa high
CEN-2Z	Poorly differentiated	SqCa high
SUNE-1-5-8F	Poorly differentiated	SqCa extremely high
SUNE-1-6-10B	Poorly differentiated	SqCa low
CEN-1	Highly differentiated	SqCa middle

Extremely high, high, middle and low refer to the degree of metastasis. SqCa, squamous cell carcinoma.

**Table II:** Comparison of KAI1/CD82 protein positive expression rates in five cell lines.

Cell line	Cells, n	Positive cells, n	Positive rate (%)^a^	χ^2^	P-value
SUNE-1-6-10B	2000	1410	70.5	-	-
CNE-1^a^	2000	814	40.7	459	4.89×10^−83^
CNE-2Z^a^	2000	803	40.2	467	1.29×10^−101^
SUNE-1^a^	2000	736	36.8	526	6.77×10^−100^
SUNE-1-5-8F^a^	2000	394	19.7	1196	6.41×10^−248^

a Compared with SUNE-1-6-10B (tumorigenesis and low metastatic), P<0.05.

**Table III:** Expression level changes of KAI1/CD82 gene in different human nasopharyngeal carcinoma cell lines.

Group	n	OD^a^
SUNE-1-6-10B	3	1594+13.95
CNE-1	3	1453+11.34
CNE-2Z	3	1326+11.78
SUNE-1	3	1314+9.09
SUNE-1-5-8F	3	1245+10.42

a Mean ± SD.

n, number of experimental repeats; OD, optical density.

**Table IV:** Association between KAI1/CD82 protein expression and patient clinical parameters.

		KAI-1/CD82 protein expression			
				
Clinical parameter	n (%)	+	−	χ^2^	P-value
Gender				0.320	0.572
Male	32 (45.7)	13	19		
Female	38 (54.3)	18	20		
Age, years				0.170	0.681
≤50	41 (58.6)	19	22		
>50	29 (41.4)	12	17		
Pathological type				0.854	1.000
Squamous cell carcinoma	69 (98.6)	32	37		
Adenocarcinoma	1 (1.4)	0	1		
T staging				0.797	0.372
T1–T2	21 (30.0)	11	10		
T3–T4	49 (70.0)	20	29		
N staging				6.157	0.013
N_0_	19 (27.1)	13	6		
N_1_	16 (22.9)	7	9		
N_2_	27 (38.6)	9	18		
N_3_	8 (11.4)	2	6		
